# *QuickStats:* Health Center Visit Rates,* by Age and Sex — United States, 2024

**DOI:** 10.15585/mmwr.mm7528a3

**Published:** 2026-07-23

**Figure:**
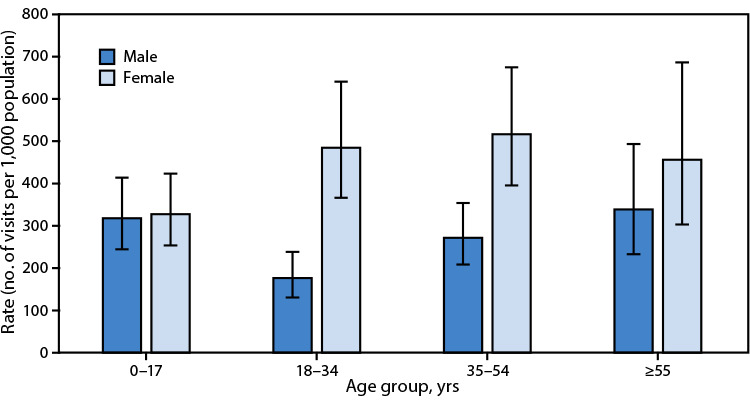
In 2024, visit rates to health centers were similar among girls (318.0 per 1,000 population) and boys (327.7) aged 0–17 years. However, visit rates were significantly higher among women aged 18–34 years (484.4) and aged 35–54 years (516.6) compared with men in the same age groups (176.5 and 271.5, respectively). The observed difference by sex was not statistically significant among adults aged ≥55 years.

**Supplementary Table:** https://stacks.cdc.gov/view/cdc/257653#tabs-3

**Source:** National Center for Health Statistics, National Ambulatory Medical Care Survey Health Center Component, 2024. National Ambulatory Medical Care Survey

